# The Genetic Basis of Dormancy and Awakening in Cutaneous Metastatic Melanoma

**DOI:** 10.3390/cancers14092104

**Published:** 2022-04-23

**Authors:** Agata Janowska, Michela Iannone, Cristian Fidanzi, Marco Romanelli, Luca Filippi, Marzia Del Re, Manuella Martins, Valentina Dini

**Affiliations:** 1 Unit of Dermatology, University of Pisa, 56126 Pisa, Italy; michela.iannone@phd.unipi.it (M.I.); cristian.fidanzi@student.unisi.it (C.F.); marco.romanelli@unipi.it (M.R.); manuella.martins@sns.it (M.M.); valentina.dini@unipi.it (V.D.); 2 Unit of Neonatology, University of Pisa, 56126 Pisa, Italy; luca.filippi@unipi.it; 3 Unit of Clinical Pharmacology and Pharmacogenetics, University of Pisa, 56126 Pisa, Italy; marzia.delre@unipi.it

**Keywords:** malignant melanoma, dormant state, clinical latency, metastatic melanoma

## Abstract

**Simple Summary:**

Advanced malignant melanoma still has a poor prognosis. Mortality is closely associated with tumor recurrence, which, as with other types of cancer, can occur after long periods of clinical remission. Clinical latency is related to the ability of residual tumor cells to persist in a dormant state, without proliferation. In this paper, we review the genetic profile of melanoma cells from their appearance, through the dormant state to their reactivation leading to metastasis. A complete genetic profile will enable the genes responsible for metastasis appearance to be identified and thus contribute to creating new therapeutical targets that keep cells in a dormant state and prevent melanoma tumor cells from spreading.

**Abstract:**

Immune dysregulation, in combination with genetic and epigenetic alterations, induces an excessive proliferation of uncontrolled melanoma cells followed by dissemination of the tumor cells to distant sites, invading organs and creating metastasis. Although immunotherapy, checkpoint inhibitors and molecular targeted therapies have been developed as treatment options for advanced melanoma, there are specific mechanisms by which cancer cells can escape treatment. One of the main factors associated with reduced response to therapy is the ability of residual tumor cells to persist in a dormant state, without proliferation. This comprehensive review aimed at understanding the genetic basis of dormancy/awakening phenomenon in metastatic melanoma will help identify the possible therapeutical strategies that might eliminate melanoma circulating tumor cells (CTCs) or keep them in the dormant state forever, thereby repressing tumor relapse and metastatic spread.

## 1. Introduction

Cutaneous metastatic melanoma is associated with 75% of skin cancer deaths and has an increasing worldwide incidence [1,2]. The course is mostly fatal with a five-year overall survival rate of 23% in stage IV patients [1]. Despite considerable progress in immune checkpoint inhibitors, as well as targeted inhibitors of *BRAF^V600E^* and *MEK* kinases, there is still widespread resistance to therapy, thus leading to disease recurrence [3]. The appearance of melanoma is due to increasing genetic and epigenetic alterations, which create an imbalance in the homeostatic signaling pathways. This then leads to excessive proliferation of uncontrolled tumor cells followed by their dissemination to distant sites, invading organs and creating metastasis. On sun-exposed skin, the primary cause of mutations is UV radiation, which increases the probability of development of cutaneous melanocytic neoplasms. Consequently, the susceptibility of a person to melanoma depends on several genetic factors that can influence the rate at which mutations are generated and fixed, for instance the ability of melanocytes to protect themselves from UV radiation by inducing tanning [4]. Genetic factors can influence the appearance and progression of melanoma. However, hereditary and non-hereditary melanomas have different somatic mutations and expression profiles. Approximately 5–12% of melanomas are hereditary and are related to the *CDKN2A* mutation, causing a defect in p14, p16 and p53- proteins related mechanisms of oncosuppression [5]. In non-hereditary melanoma, multiple molecular pathways are implicated. The most relevant are the *mitogen-activated protein kinase (MAPK)/ extracellular signal-regulated kinase (ERK)* pathway (composed of *TKR, RAS, B-RAF, MEK, ERK and NF1*), which are involved in cell proliferation, apoptosis, differentiation, adhesion, and mobility, and the *PI3K/AKT* pathway responsible for cell survival and regulation of apoptosis [6]. Interestingly, around 80% of benign nevi have the *V600 BRAF* mutation. However, *BRAF* mutation by itself is not sufficient to generate a malignant phenotype, and more than one mutation is needed to transform normal melanocytes into a malignant tumor. It is estimated that 50% of melanomas have a *B-RAF* mutation; another 15–20% have an *NRAS* mutation, and 2% have a *c-KIT* mutation (mucosal melanoma) [7]. As previously mentioned, UV radiation plays a key role in melanocyte stimulation to develop melanoma by direct stimulation of *MAPK* signaling. The management of patients and their life expectancy can be improved by understanding the key steps in the metastatic cascade along with the mechanisms behind the development of resistance to therapies, related to a non-mutational enrichment of a subpopulation of cancer stem cells that are intrinsically refractory to the effects of anticancer drugs [8]. Melanoma cells have a high degree of plasticity due to a cellular embryogenetic program of upregulation of mesenchymal markers with a downregulation of epithelial markers called epithelial-mesenchymal transition (ETM) and epigenetic induction. An integrative reciprocity of ETM, dormancy, metabolic reprogramming, and the tumor microenvironment orchestrates phenotype-switching from a melanocyte-like and hyperdifferentiated/pigmented state to a starved melanoma cell (SMC), intermediate melanoma cell, neural crest stem cell (NCSC) to undifferentiated cells. Phenotype-switching is based on the relative expression of transcription factors, such as the microphtalmia transcription factor (MITF) and SOX10. Undifferentiated melanoma cells detach from the basal lamina and undergo further dedifferentiation involving cadherin, integrin, laminin and desmoglein isoforms, with subsequent transendothelial migration and invasion. Dissemination is then facilitated by increased tolerability of oxidative stress through the development of the mitochondrial metabolism with metabolic symbiosis between hypoxic and aerobic melanoma cells and with the stromal neighborhood through tumor-secreted soluble factors and extracellular vesicles called exosomes. The creation of a cancer-friendly secondary ECM through exosomes leads to organotropism [9]. The majority of disseminated melanoma cells die as a result of immunosurveillance mechanisms mediated by CD8+ T cells. Only a fraction of cells has the potential for early recurrence with early metastasis or the induction of dormancy and late metastasis [10]. Breslow thickness associated with high proliferative activity represented by the Ki-67 index (the result of the increased expression of proto-oncogenes and decreased expression of putative tumor suppressor genes) are currently the most important prognostic factors for invasive melanoma [11]. In this paper, we present a complete genetic profile of melanoma cells from their dormant state to their reactivation. A complete genetic profile enables new therapeutic targets to be identified, which are capable of keeping cells in a dormant state and preventing late metastasis.

## 2. Gold Standard Melanoma Markers

In physiological conditions, melanocytes divide less than twice per year presenting a low proliferative ratio. However, the complex combination of exogenous and endogenous factors may rapidly increase the melanocytic division rate, increasing the number of malignant melanocytic cells that lead to metastasis appearance and melanoma progression [12]. Due to its cytomorphological variants, melanoma can resemble different tumors such as carcinomas, neuroendocrine tumors, sarcomas, lymphomas and germ cell tumors. This increases the difficulty in establishing an accurate diagnosis, and thus melanocytic markers are crucial in the diagnosis of metastatic melanoma. For this stage, there are several markers for melanoma-initiation, such as MAGE proteins, GalNAc-T, NG2, CSPG4, ABCB5, MAGEA3, PAX3, TGFB2, TYR2, Human Melanoma Black-45 (HMB-45), Melan-A, tyrosinase, and S100 [7,8,9,10,11,12,13,14,15,16,17,18]. HMB-45, also known as Pmel or gp100, is found in pre-melanosomal vesicles and is believed to be fundamental for the polymerization of eumelanin. Interestingly, levels of HMB-45 are lower in metastatic melanoma compared to primary lesions, and its specificity is lower for malignant melanoma in sentinel lymph nodes compared to other markers, such as Melan-A [13]. The progression of melanoma and metastasis appearance is associated with the spread of melanoma circulating tumor cells (CTCs), which are shed into the bloodstream from either the primary or the metastatic cancer niche [19]. Melanoma CTCs are identified by specific markers. Melanoma specific cell adhesion molecules (MCAM) have been used as the first level for selecting melanoma circulating cells. After this initial screening of CTCs, markers such as melanoma-associated chondroitin sulfate proteoglycan (MCSP), chondroitin-surface proteoglycan 4 (CSGP4), and human high molecular weight melanoma-associated antigen (HMW-MAA) have been used in the immunostaining panel [20,21,22].

## 3. Dormancy

Surgery is the standard procedure used to treat melanoma aimed at removing the cancer cells. Unfortunately, even after completion of treatment, patients may still carry cancer cells that remain *silent* for long periods of time, which is called “cell dormancy”. Dormant cells can be defined as “stem cells in a quiescent state” due to reversible arrest of the cell cycle through increased expression of metastatic suppressor genes and complex molecular signaling cascades [23]. As they have a short lifespan, animal models facilitate the understanding of tumor dormancy biology in vivo. In order to fully understand the dynamics of disease spread or response to specific therapies, it is crucial to study the interactions between cancer cells and the surrounding microenvironment [24]. For this purpose, high-resolution imaging of fluorescently labeled melanoma cells has been used in a semi-transparent zebrafish model. This model provided new insights into the metastasis-promoting factor keratinocyte-derived endothelin 3 (EDN3) and its role in the switch of melanoma cells between invasive and proliferative states [25].

**Figure 1:**
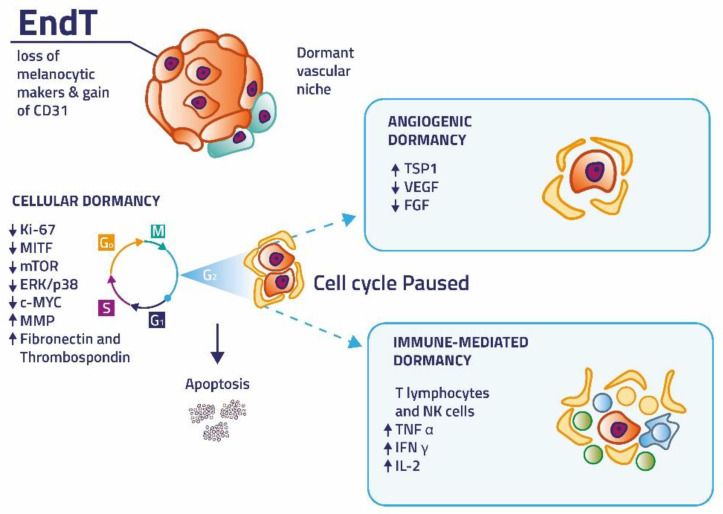
The main factors contributing to the process of melanoma dormancy: cellular dormancy, angiogenic dormancy and immune-mediated dormancy.

The intricate combination of events enables these cells to release specific factors that restrain their growth-signaling pathways and help them escape immune surveillance and chemotherapy. Dormant melanoma cells act together by controlling the immune regulation, causing impaired angiogenesis, and arresting the cell cycle [26,27]. Clinically, tumor dormancy depends on at least three elements. The first is called cellular dormancy, in which single tumor cells persist in a quiescent, slowly dividing state (with a low expression of Ki-67). The second is called angiogenic dormancy, in which lack of vascularization holds the growth of micrometastasis in check and promotes apoptosis. The final element is immune-mediated dormancy, where the immune system continues to limit the tumor population [4]. Then, specific and as yet unknown processes, may awake dormant micrometastasis and these cells become active again. Once activated, tumor cells expand, proliferate and form metastasis at a distant site from the primary tumor, leading to patient morbidity and mortality [28]. A possible prevalence of apoptotic phenomena on proliferation, or a strong anti-tumor immune response associated with deficient stromal receptivity, including any angiogenesis defects, mediate tumor regression [11]. Revealing the main factors contributing to the process of tumor dormancy will help identify new genetic targets to be used in the clinic as therapeutical approaches to stop the progression of melanoma (Figure 1).

### 3.1. The First Step: Cellular Dormancy

The activation of quiescence processes associated with cellular dormancy is characterized by the development of KI-67 negative cells without signals of apoptosis. The in vivo and in vitro analyses of mouse models have contributed to understanding the numerous mechanisms, such as absence/alterations of crucial signaling pathways or input from new signals in the microenvironment, through which cellular dormancy takes place [29]. One of the most important processes is phenotypic plasticity with differing MITF expression. MITF represents a melanocytic lineage-specific transcription factor, which has a function that is directly correlated to malignant melanoma [30]. Several studies have correlated the level of MITF expression with differentiation/proliferation (MITF overexpression) or quiescence (MITF low levels) [31]. Therefore, low MITF levels are present in populations with stem cell-like behavior and p-27 dependent cell cycle arrest. This phenotypic plasticity should be considered when studying melanoma dormancy as a specific pathway in that niche of cells might disable or activate the switch, thus regulating melanoma cell invasiveness [32]. The levels of MITF are inversely correlated with those of several activated receptor tyrosine kinases, most frequently AXL. A low MITF/AXL ratio predicts early resistance to multiple targeted drugs in melanoma, further linking dormancy mediating signals to melanoma plasticity [33].

Another key pathway in cellular dormancy is the downregulation of *mTOR* signaling, via altered *PI3K/AKT, PTEN* inactivation, altered *LKB1/AMPK* cascade, and autophagy induction [34]. In mice models with melanocyte-specific PTEN deletion and *BRAF^V600E^* allelic mutation, the rapid development of melanoma forms characterized by visceral invasion has been observed [35]. PTEN loss has been shown to stimulate an increased fibronectin production, increased *PI3K/AKT*-mediated survival signaling, and simultaneous inhibition of the *PI3K* pathway, thus impairing the response to *BRAF* inhibitor therapy (*BRAFi*) and promoting the induction of treatment resistance [36].

Regarding the *AKT* pathway, a decreased activity in in vitro models was associated with the quiescence of melanoma stem-like cell subpopulations, while the overactivation of the pathway was shown to awaken cells from *BRAF-*induced senescence [37].

In addition, *BRAF^V600E^* allelic mutation induces the inactivation of *LKB1/AMPK* signaling through *LKB1* inhibition by *ERK* and *RSK,* thus compromising the ability of *LKB1* to bind and activate *AMPK*. This results in melanoma cell proliferation and anchorage-independent cell growth although in melanocytic nevi, it is insufficient for complete progression to melanoma [38,39].

Data on melanoma have been confirmed in genetically engineered mice with the development of invasive melanoma after the inactivation of *LKB1* in a *K-Ras-*driven melanoma model and by evidence of endothelial transdifferentiation in *BRAF^V600E^*-metastatic biopsies from the human lung, brain, and small intestine [40,41].

The *MAPK/ERK* pathway is also crucially important for determining whether cancer cells proliferate or enter a state of dormancy [42]. Once this pathway is activated, it induces a quick and vigorous nuclear translocation with the phosphorylation of transcription factors so*B-RAF*, and *N/K-RAS* transformed-cancer cells start to proliferate [43]. However, it seems that all these events change the balance between p38 and ERK activities, in which a low *ERK/p38* ratio is associated with tumor growth arrest and dormancy, whereas a high *ERK/p38* ratio favors tumor growth [44,45]. In fact, a low *ERK/p38* ratio downregulates proliferation-associated transcription factors, such as *FOXM1* and *c-Jun18,* but upregulates cell cycle inhibitors such as cyclin-dependent kinase inhibitors p27 and p21, as well as nuclear receptor subfamily 2 group F member 1 (NR2F1) and basic helix-loop-helix protein 3 (BHLHB3) [46,47]. Cell cycle arrest mediates chemoresistance and the inhibition of aerobic glycolysis in dormant cells [48].

The capacity of dormant cells to survive is related to the switch to the mitochondrial oxidative phenotype with the use of glutamine or fatty acids oxidized for energy production and a subsequent growth advantage under metabolic stress conditions. The mitochondrial metabolism is therefore crucial for metastatization and for resistance to *BRAF* and *MEK* inhibitors. These in vitro data highlight the importance of combining in vivo mitochondrial and *MAPK/ERK* pathway inhibition to improve the efficacy of therapy [49].

The transforming growth factor-β (TGF-β) is another superfamily linked to tumor dormancy. In normal epithelial cells, *TGFβ* has cytostatic activity, while in melanoma cells it promotes peri-tumoral angiogenesis, cell migration, immune escape, and dissemination to metastatic sites [50]. Research carried out in vitro and in a mouse model of human melanoma bone metastasis found that blocking the *TGF-β* can prevent the development of bone metastases and decrease the progression of established osteolytic lesions [51]. The bind on tumor cell membranes of *TGF-β2* to its receptors, TGF-β Receptor-I (TGF-β-R1) and TGF-β-R3, leads to the stimulation of p38 and consequently the downregulation of *ERK* signaling. The signaling cascade activates *Smad1/5*, which augments *DEC2/SHARP1* levels as well as p27, but inhibits cyclin-dependent kinase 4 (CDK4), bringing the tumor cells into a quiescent state [52].

Early dissemination of melanoma cells may occur before they lose the ability to respond to *TGF-β* dependent growth-inhibitory signals, and the plasticity of melanoma cells to switch in vivo between the *TGF-β* responsive and refractory state could also explain the ability to enter the dormant state [53]. In addition, long-term *TGFβ* exposure can induce a dedifferentiated EMT-like state with the melanoma invasive phenotype characterized by MITF downregulation and mesenchymal marker upregulation, thus driving the invasiveness of the melanoma [54]. Subsequently, *TGF-β* expression correlates with dedifferentiated melanoma cells, and in association with the increased expression of *ZEB2/SLUG/MITF*, may mediate phenotype-switching from melanocyte-like and hyperdifferentiated/pigmented state to SMC, intermediate melanoma cells, NCS to undifferentiated cells [55,56].

To complete the dormancy profile, it is crucial to highlight the interactions with ECM factors. The contact of melanoma cells with fibrillar collagen induces cell cycle arrest with dormancy in in vitro models, thus preventing ß1 integrins from clustering with high levels of p27 mRNA and protein. The crucial importance of the tumor microenvironment has been confirmed by in vivo and in vitro models studying minimal residual disease (defined between two and four mm in size) [57].

Tissue stiffness and matrix composition are involved in the response of melanoma cells to the *BRAF* inhibitor *(BRAFi*) through melanoma-associated fibroblasts (MAFs). MAFs are involved in the increase in fibronectin, tenascin c, and thrombospondin and in reactivation of *ERK* via *ß1 integrin/FAK* signaling. The combination of *BRAF*i with *FAK* inhibitors (*FAK*i) results in a prolonged response with increased tumor stability from 50 days (in cases treated with *BRAF*i alone) to 120 days in cases of combination therapy. Another important finding is the stable Ki67 levels during *BRAF*i treatment (reflecting a balance between proliferation and apoptosis) versus decreased levels during combination treatment. This confirms a mechanism of control of tumor growth by dormancy induction, which prevents the accumulation of genetic alterations that drive the recurrence of resistant tumors [58].

Another 3D ECM model, derived from fibroblasts isolated in the biopsies of patients with metastatic melanoma, demonstrated the existence of matrix-mediated drug resistance to *BRAF*-targeted therapy, which is related to the activation of the tyrosine kinase receptors for collagens, i.e., discoidin domain receptors 1 and 2 (DDR1–2) inducing physical and structural changes in MAFs [59].

Other important growth regulators in melanoma cells are metalloproteases (MMPs), which are enzymes that degrade ECM and are associated with tumor progression. Studies in vitro investigated the importance of MMP14 in melanoma progression and showed that the increase in collagen XIV in the ECM produced by fibroblasts deprived of MMP14 inhibited proliferation, migration and adhesion of melanoma cells [60]. MMP-9 also plays an important role in reshaping the premetastatic niche and facilitating colonization of CTCs [61].

### 3.2. Angiogenic Dormancy

Angiogenic dormancy is defined as “a period of balance between proliferation and cell death as a consequence of oxygen and nutrient deprivation” with a stable vascularization [62]. The variation in the balance between pro-angiogenic and antiangiogenic factors with angiogenic switch and escape from angiogenic dormancy, would appear to promote tumor or metastatic outgrowth. Data were confirmed by several mouse models, where the inhibition of angiogenesis by angiostatin induced dormancy of metastases, thus inhibiting the growth of three human and three murine primary carcinomas [63]. Angiogenetic dormancy is also associated with an overexpression of angiogenesis inhibitors such as thrombospondin 1 (TSP-1), which decreases *VEGF* and *FGF* expression. Melanoma primary tumor secretes TSP-1, which inhibits lung metastasis and decreases angiogenesis [21,64]. In addition, after radiation therapy, the administration of TSP-1 prevents the outgrowth of dormant macrometastasis [65,66]. Melanoma susceptibility to microenvironmental growth-inhibitory signals was further confirmed in tumor-bearing mice models: following a Th1 viral infection, CD4+ andCD8+ T cells release TSP-1 with subsequent angiogenetic inhibition and a reduction in tumor growth [67]. TSP-1 levels were also inversely correlated with MITF expression and pigmentation, suggesting the oncogenic role of this factor correlated with de-differentiated EMT-like melanoma phenotypes [68].

### 3.3. Immune-Mediated Dormancy

Immune-mediated dormancy transmits melanoma in transplanted individuals with rapid growth recovery in an immunosuppressed host. The control of tumor growth by the immune system is called ‘immunoediting’ and takes place via immunosurveillance, dormancy and escape. Immunosurveillance is based on the immune system’s ability to eliminate transformed cells, and immune-mediate dormancy is achieved through cytostatic and cytolytic immune activity resulting in the blockage of tumor growth. Awakening from dormancy is achieved through the immune responses’ attenuation in cancer growth [69].

Mouse models have clarified the role of innate and adaptive immunity in immune mediate dormancy. Growth arrest is achieved by the killing of tumor cells by T lymphocytes and NK cells. T cells and innate immune cells also produce IFN-γ, which inhibits angiogenesis inducing angiostatic cytokines (CXCL9/CXCL10) and in combination with TNF-α, blocks cell cycle progression in neoplastic cells [67,70,71,72]. Similarly to IFN-γ, IL-2 also regulates adaptive immune responses by mediating the growth and differentiation of T cells and NK cells and eliciting immunosuppressing functions of Treg cells. In a small group of advanced melanoma patients, the use of IL-2 as adjuvant therapy showed excellent results in suppressing metastases [72,73]. Studies on melanoma cells showed that IL-2 production increases the amount of interferon-γ-expressing CD8 T and natural killer cells in the tumor mass enhance *Foxp3*(+) CD4(+) regulatory T cells and anti-inflammatory cytokines such as IL-10, and also favor the expression of vascular cell adhesion molecule 1 on tumor vessels. These responses, which are decisive in the development of tumor dormancy, were largely absent in interferon-γ knockout mice [74].

Assuming that the duration of *BRAF*-inhibition is short, the potential association with immune-checkpoint therapies that modulate the immune system could improve the in vivo management of patients with advanced melanoma [49,75].

## 4. Awakening

### Activation of Cells

Dormant melanoma cells can reside for years at their dormant niches and potentially transdifferentiate to endothelial cells by endothelial transition (EndT), where melanocytic markers are lost and the endothelial marker CD31 is gained. There are several factors and signaling cascades that can help the awakening of dormant cells, including mechanisms related to surgical resection, with the creation of the environment that facilitates tumor metastasis [11].

One of these factors is β-1 integrin signaling. The inhibition of β-1integrin signaling leads to tumor growth arrest and apoptosis. On the other hand, the reactivation of the signaling promotes cell polarity, motility, differentiation, proliferation and survival [76].

In addition, chronic inflammation could be one of the main mechanisms responsible for awakening dormant cancer cells. Chronic inflammation leads to increased angiogenesis, which delivers oxygen and nutrients to cancer cells, thus promoting their growth, and consequently interrupting the dormant state of those cells. Inflammatory cytokines and epigenetic alterations in tumor suppressor genes can also increase carcinogenesis.

Another key factor for the awakening of tumor cells is *TGF-β* signaling. In the early stages of carcinogenesis, *TGF-β* upregulates cyclin-dependent kinase (CDK) inhibitors, such as p21 and p15, and, at the same time, represses *c-MYC*, which is an inhibitor of differentiation (ID) family members. Together with *SMAD* signaling pathways, *TGF-β* is also crucial for the regulation of numerous pro-apoptotic genes. *TGF-β* is therefore regarded as a tumor suppressor. However, when melanoma cells proliferate, they secrete high levels of *TGF-β*, which in turn acts on tumor cells and TME, contributing to angiogenesis, tumor cell proliferation, and metastasis formation [77].

The role of the urokinase-type plasminogen activator (uPA) and its receptor (uPAR) in cancer progression help explain the awakening of malignant melanoma stem cells (MMSCs). The binding of uPA to its receptor (uPAr) triggers a proteolytic cascade, which leads to the activation of *VEGF, EGF, FGF-2* and *TGF-β*, as well as *β1-integrins* [78]. The interaction of uPAR to integrins, induces the mitogenic *Raf-MEK-ERK signaling* pathways, which are important for melanoma dormancy as well as tumor cell migration, thus suggesting a possible correlation between these two events [45] In fact, the inhibition of uPAR, integrin β1 or *EGFR*, either alone or combined, prompts dormancy and tumor suppression [79]. It is worth highlighting that progression of melanoma was impaired in uPA-deficient mice, but pancreatic cancer progression was not affected when uPA was blocked in a transgenic mice model of RIP-Tag2. Thus, this indicates that uPA system mediation in tumorigenesis varies among different cancer types [80,81].

The pathway of the receptor activator of *NF-kB (RANK*)/receptor activator of *NF-kB* ligand (*RANKL*) is crucial for the migration and metastasis of activated tumor cells [82]. Peripheral blood, primary tumor or metastases at an advanced stage (IV) that present *RANK*^+^-melanoma cells, co-express *ABCB5*, *MART-1* and *CD133* [82]. These genes are common markers of melanoma initiating-cells, and their presence together with *RANK* identifies the tumor stem cell-like phenotype. Interestingly, there are more *RANK-*expressing melanoma cells in metastasis compared to primary tumors, and they are more common amongst CTCs than in solid tumors [83]. One study also indicates that melanoma cells expressing *RANK* show a reduced *Ki-67* proliferation in patients at stage IV compared to melanoma cells at stage I, thus confirming the dormancy state. These same cells also induce tumors in immunodeficient mice [82]. Analyzing the specificity of tumor-reactive T-cell clones derived from a patient with metastatic cutaneous melanoma, a tumor-associated antigen was recently identified, known as PRAME (preferentially expressed antigen in melanoma). PRAME is expressed in cutaneous and ocular melanoma as well as in various non-melanocytic malignant neoplasms. Normal healthy tissues are not known to express PRAME except for the testis, ovary, placenta, adrenals, and endometrium, thus, its expression in melanocytes is a strong indicator of metastatic melanoma [84].

The interaction with ECM is also critical for cell awakening. The proteolytic degradation of collagen fibrils, together with the altered expression of cell adhesion molecules (CAMs), such as cadherin, integrin, immunoglobulin, and selectin protein families enable tumor cells to escape dormancy with the reactivation of growth and metastasization. Selectins expressed on the surrounding microenvironment cells are associated with the risk of metastasis [85].

A number of cellular stress factors such as hypoxia, nutrient deprivation or inducers of reactive oxygen species (ROS) through important intracellular mediators (such as *NFKB* and *P53*) or oncogenes (such as *RAS* or *MYC*) are important stimuli of angiogenic signaling with the subsequent escape from angiogenic dormancy and development of highly invasive tumors [86]. Targeting the angiogenic switch with antiangiogenic therapeutics has become an important therapeutic strategy, although it is also affected by transient results and treatment resistance [87]. These results could be related to the de novo formation of vascular networks by tumor cells independently of *VEGF* and mediated by the vascular cell adhesion molecule PECAM1 [88]. The ability of melanoma cells to metastasize independently of *VEGF* and angiogenetic factors was confirmed by an in vivo study on mouse models. Melanocytic cells used preexisting brain vasculature to spread and were detected strictly in perivascular sites, while dormant cells moved steadily along blood vessels where oxygen and nutrients were abundant, thus suggesting an important role of the vascular system in angiogenetic dormancy [89].

Other relevant factors in dormancy escape are the increased levels of interferon gamma *IFN-γ* and re-expression of glucocorticoid-induced leucine zipper (*GILZ).* *IFN-γ* modulates CD4+-Tcell viability and the expression of immune-checkpoint molecules and changes in tumor-associated macrophage polarization with the subsequent stimulation of cell proliferation and angiogenesis, and the development of macrometastasis [90,91].

*GILZ* inactivating *FOXO3A* and its downstream target, *p21CIP1*, re-enters dormant tumor cells within the cell cycle, resulting in faster and more aggressive melanoma relapse [92]

The main factors involved in the melanoma dormancy/awakening phenomenon are summarized in Table 1.

## 5. Therapeutic Implications

Tumor dormancy is crucial for cancer cells to survive in a new environment, acquire additional mutations, and induce metastasis formation. Consequently, these cells escape the immune system and are resistant to cancer therapy. Various strategies are trying metastasis formation and melanoma progression, including keeping cancer cells in a dormant state, and targeting signaling molecules and microenvironments. A key issue related to keeping tumor cells in dormant state is the need to carry out life-long treatment. A completely different approach is to activate dormant cells in order to improve the efficacy of anti-proliferative therapy [93]

In a study on xenograft models to stimulate awakening in dormant cells, thus making them more susceptible to treatment, methotrexate was reported to favor the phenotypic switch from a *MITF* low to an *MITF* high state, thus sensitizing melanoma cells to a tyrosinase-processed antifolate prodrug [94]. The risk of using these treatments that have been validated in mouse models in vivo is the loss of tumor growth control with consequently worse patient outcomes [95,96].

Therapeutic strategies for eradicating dormant cells by impairing major survival pathways or mechanisms that mediate resistance to therapy are more promising. Many of the most interesting new drugs are related to immune-mediated dormancy. Enhancement of the T-cell antitumor response is mediated by blocking the *Lymphocyte activation gene-3 (LAG-3), glucocorticoid-induced TNF receptor (GITR)* and T cell immunoreceptor with immunoglobulin and ITIM domain (*TIGIT*), with subsequent T-cell effectors and NK cell proliferation, release of pro-inflammatory cytokine, and inhibition of Treg cells resulting in a more effective eradication of dormant cancer cells [97].

IL-2 agonists are also promising molecules with a similar action to the above [71,97].

Regarding new targeted therapy opportunities, there are several molecules involved in cellular dormancy, such as new *MEK* (Bimitinib/Pimasertib/FNC-59), *RAF* inhibitors (LXH254), *Cyclin-dependent kinase (CDKs*) inhibitors, *MAP/ERK* pathway inhibitors (Ulixertinib, LY3214996 and ASN007), *Histone Deacetylase inhibitor* (Vorinostat/Domatinostat and Entinostat), *Indoleamine-2,3-dioxygenase (IDO)* inhibitors (Epacadostat, BMS-986205, Indoximod), *Receptor Tyrosine Kinase Axl inhibitors* (Bemcentinib), *Ataxia telangiectasia* and *Rad3-related protein kinase inhibitors* (Ceralasertib), and *Toll-like Receptor (TLR) 9* Agonist (Tilsotolimod, CMP-001, SD-101) [97].

Regarding angiogenetic dormancy, the effects of angiogenesis inhibitors are currently under investigation. The *VEGF*-blocker Bevacizumab did not appear to have clinical benefits as a first-line therapy in advanced melanoma, either in monotherapy or in combination with cytotoxic drugs, such as carboplatin and paclitaxel. One promising strategy is to combine antiangiogenic therapy with ipilimumab, a CTLA4 monoclonal antibody [98]. Other molecules being used in trials are Lenvatinib and Axitinib.

β-adrenergic(β-A) arrangement also plays a key role in regulating the immune system [95,96,97,98,99,100,101]. β3-adrenoceptors *(β3-AR)* modulate the immune response by controlling several factors that are crucial for immune cell subpopulation homeostasis. In fact, β3-AR are expressed in human and mouse melanoma cells as well as in cells of its tumor microenvironment. In addition, their levels increase when all these cells are exposed to hypoxia, and they are actively involved in proliferation (A) and vascularization of melanoma, through the pathway vascular endothelial growth factor (*VEGF)*-nitric oxide [102,103].

*β3-AR* up-regulation also appears to be significantly related to stemness, with the induction of mitochondrial dormancy responsible for a metabolic adaption used to survive in a hypoxic environment and to reduce ROS production, to promote chemoresistance, and lastly to induce an immune-tolerant halo around cancer. The hypoxia-induced up-regulation of these receptors appears to be exploited by cancer to grow in a non-hospital environment. The *β3-AR* signaling pathway could thus be directly associated with melanoma progression and, by targeting the specific genes of this pathway, cells could be kept in a dormant state and thus metastasis would be prevented [104].

Other promising therapeutic strategies include vaccines and the targeted therapy of tumor antigens normally expressed in germ cells, which are expressed again in tumor cells, named cancer testis antigens (CTAc) [105]. Among the most important CTAs in melanoma are NY-ESO-1 antigens, which are expressed by 24% of vertically growing melanomas.

A clinical trial using recombinant vaccinia-NY-ESO-1 antigen showed an objective response rate of 14%, a mixed response of 5%, and disease stabilization of 52%, amounting to a clinical benefit rate of 72% in melanoma patients [106,107].

PRAME is another attractive member for immunotherapy of the CTA family. One study showed that around 87% of metastatic melanomas expressed PRAME in a diffuse homogenous pattern, with an additional 5% of cases (total of 92% of cases) showing at least focal immunoreactivity. The frequency of PRAME expression in melanoma-based immunohistochemical detection is also similar to the reported frequency of 91%, which was based on mRNA studies.

PRAME could thus be used as a target for immunotherapy in melanoma patients [86]. A dose-escalation phase I/II study (NCT01149343) with recombinant PRAME protein with the AS15 immunostimulant in 66 patients with advanced PRAME-positive melanoma showed an acceptable safety profile and induction of humoral and cellular immune response. Further studies are underway to validate these results [105].

Regarding cancer vaccines, the aim is to stimulate tumor-specific immune responses with long-term memory to prevent relapse or metastasis, with a low risk of overall toxicity.

Ongoing clinical trials for the treatment of metastatic or unresectable advanced cutaneous melanoma have explored the effectiveness and safety of the combination strategies by adding other immune-modulating agents to the standard of care for a more aggressive management of untreated metastatic melanoma or targeted therapies in pre-treated patients who have developed resistance [99].

## 6. Conclusions

The mechanisms involved in tumor cell dormancy and awakening are extremely complex and heterogeneous. Many of the studies aimed at understanding these mechanisms are based on in vitro studies that do not fully reflect in vivo conditions. It is important to continue in vivo research in order to confirm the available data and validate the therapeutic possibilities on a large scale. The combination of general immune mechanisms with targeted therapy, taking into account intra-tumor heterogeneity and cellular plasticity, may be the solution to eradicating tumors and their dormant lesions, thereby preventing phenotype switching towards treatment-resistant subpopulations and prolonging the patient’s survival.

## Figures and Tables

**Table 1:** Main factors involved in melanoma dormancy/awakening.

Factors	Mechanisms	References
MITF (Microphthalmia-associated transcription factor)	Melanocytic lineage-specific transcription factor. Low levels are associated with quiescence	[30,31,32,33]
mTOR (Mammalian target of rapamycin)	Its downregulation causes quiescence	[34,35,36,37,38,39,40,41]
MAPK/ERK pathway	Its alteration causes quiescence: a specifically low *ERK/p38* ratio is associated with tumor growth arrest and dormancy	[42,43,44,45,46,47,48]
TGF-β (Transforming Growth Factor-β)	The binding of TGF-β2 on tumor cell membranes to its receptors, TGF-β Receptor-I (TGF-β-R1) and TGF-β-R3, leads to the stimulation of p38 and consequently downregulation of ERK signaling. These mechanisms keep the tumor cells in a quiescent state. However, under certain conditions, TGF-β, may contribute to angiogenesis, tumor cell proliferation, and metastasis formation.	[50,51,52,53,54,55,56,77]
MAFs (melanoma-associated fibroblasts)	Induce dormancy through increase in fibronectin, tenascin c, and thrombospondin and the reactivation of ERK via ß1 integrin/FAK signaling.	[58,59]
MMP (metalloprotease)	Some metalloproteases, such as MMP14 and MMP9, have an inhibitory action on proliferation, migration and adhesion of melanoma cells.	[60,61]
TSP-1 (thrombospondin 1)	Reducing VEGF and FGF expression causes angiogenetic dormancy	[21,65,66,67]
IFN-γ	Produced by T lymphocytes and innate immunity cells, it inhibits angiogenesis inducting angiostatic cytokines (CXCL9/CXCL10). IFN-γ can also stimulate cell proliferation, angiogenesis and therefore the development of macrometastases.	[70,71,72,92,93]
IL-2	Increases the amount of interferon-γ-expressing CD8 T and natural killer cells, enhances Foxp3(+) CD4(+) regulatory T cells and anti-inflammatory cytokines such as IL-10, and also favors the expression of vascular cell adhesion molecule 1 on tumor vessels; induces tumor quiescence.	[73,74,75]
β-1 integrin	Promotes cell polarity, motility, differentiation, proliferation and survival. Inhibition of this signal, on the other hand, results in the arrest of tumor growth and apoptosis.	[78]
uPA (Urokinase-Type Plasminogen Activator)	Activation of the uPAR receptor triggers a proteolytic cascade, which leads to the activation of VEGF, EGF, FGF-2, TGF-β, and β1-integrin resulting in cell proliferation.	[79,80,81,82]
RANK/RANKL	RANK-expressing melanoma cells are more common in metastasis compared with primary tumors, and are more common amongst CTCs than in solid tumors	[83,84,85]
PRAME (preferentially expressed antigen in melanoma)	Its expression in melanocytes serves as a strong indicator of metastatic melanoma.	[86]
ECM (extracellular matrix)	The contact of melanoma cells with fibrillar collagen induces cell cycle arrest with dormancy in in vitro models, by preventing ß1 integrins from clustering with high levels of p27 mRNA and protein. The proteolytic degradation of collagen fibrils, together with altered expression of cell adhesion molecules (CAMs), enable tumor cells to escape from dormancy.	[55,84]
Cellular Stress Factors	Hypoxia, nutrient deprivation or inducers of reactive oxygen species (ROS) through important intracellular mediators (such as NFKB and P53) or oncogenes (such as RAS or MYC) are important stimuli of angiogenic signaling.	[85]
PECAM1 (Platelet endothelial cell adhesion molecule-1)	Induces VEGF-independent neoangiogenesis.	[87]
GILZ (glucocorticoid-induced leucine zipper)	Inactivating FOXO3A and its downstream target, p21CIP1 re-enters dormant tumor cells into the cell cycle.	[94]
